# The Lyapunov spectra of quantum thermalisation

**DOI:** 10.1038/s41467-019-10336-4

**Published:** 2019-06-20

**Authors:** A. Hallam, J. G. Morley, A. G. Green

**Affiliations:** London Centre for Nanotechnology, University College London, Gordon St., London, WC1H 0AH UK

**Keywords:** Condensed-matter physics, Statistical physics, thermodynamics and nonlinear dynamics

## Abstract

Thermalisation in closed quantum systems occurs through a process of dephasing due to parts of the system outside of the window of observation, gradually revealing the underlying thermal nature of eigenstates. In contrast, closed classical systems thermalize due to dynamical chaos. We demonstrate a deep link between these processes. Projecting quantum dynamics onto variational states using the time-dependent variational principle, results in classical chaotic Hamiltonian dynamics. We study an infinite spin chain in two ways—using the matrix product state ansatz for the wavefunction and for the thermofield purification of the density matrix—and extract the full Lyapunov spectrum of the resulting dynamics. We show that the entanglement growth rate is related to the Kolmogorov–Sinai entropy of dynamics projected onto states with appropriate entanglement, extending previous results about initial entanglement growth to all times. The Lyapunov spectra for thermofield descriptions of thermalizing systems show a remarkable semi-circular distribution.

## Abstract (web summary)

The relationship between thermalisation of classical system via dynamical chaos and the one of closed quantum systems is far from totally understood. Here, the authors shed light on this connection by projecting quantum dynamics to classical chaotic ones and determining its Lyapunov spectrum.

## Introduction

The extra information required to specify a pure quantum state compared with that required for a classical or thermal state underpins many of the apparent paradoxes of quantum mechanics^1^. These may be profoundly philosophical, such as when attempting to apply quantum mechanics to the whole universe, e.g. the black-hole information paradox, and the very long-scale entanglement implied by the origin of microwave background anisotropy in zero-point fluctuations^2^. Whilst there are fewer philosophical difficulties in the description of finite quantum systems, there are practical consequences.

Accurate numerical description of a quantum system evolving from a weakly entangled initial state requires an exponentially growing number of parameters. The eigenstate thermalisation hypothesis implies that, beyond a certain point in time, an accurate representation of this dynamics should require a reducing number of parameters. The eigenstate thermalisation hypothesis^3–5^ has made great strides in demonstrating how thermal correlations present in local observations of eigenstates are revealed through a process of dephasing due to entanglement with regions of the system not directly under observation. The ultimate consequence is that late-time, local observations are characterised by just the energy density. The increase and then reduction of parameters required to accurately describe a thermalising quantum system is akin to the Page curve^6,7^ for the entanglement entropy of partitions of a system. The Page curve appears in the context of the black hole information paradox. Its appearance here is for similar reasons, except that the horizon for observations is imposed by hand and does not evaporate.

Quantum chaos^8^ provides a link between classical thermalisation—which proceeds via dynamical chaos—and quantum thermalisation. Studying few particle systems whose classical limit is chaotic, or many-body systems whose many particle dynamics is dominated by single-particle chaos, has lead to great insights into the relationship between chaos and thermalisation^9–15^. It is problematic to extend this to fundamentally many-body systems—such as spin chains—that have no clear semiclassical limit. Fortunately, the underlying chaos is reflected in the spectral statistics, which provides a measure that can be extended to many-body systems^16–20^ and is thoroughly accounted for by random matrix theory^21,22^. However, the link between quantum and classical chaos in many-body systems remains unclear. Operator spreading as quantified by the out-of-time ordered correlator^23^ is often related to the classical Lyapunov in its initial exponential growth. However, such a behaviour and identification is only found when there is a clear semiclassical limit. The link between quantum and classical chaos in many-body systems remains unclear.

Here, we demonstrate a new way to analyse quantum thermalisation that extends the connection between classical and quantum thermalisation to fundamentally many-body systems. The central idea is to project the quantum dynamics onto effective classical, Hamiltonian dynamics on a class of different variational manifolds^24,25^. Thermalisation in these classical systems occurs via dynamical chaos^26–28^. Every dynamical mode of the system is chaotic, revealed on timescales given by the inverse of its corresponding Lyapunov exponent. This distribution of timescales is quantified by the Lyapunov spectrum. We apply this reasoning to a translationally invariant spin chain, a system over which we have analytical and numerical control using matrix product state (MPS) representation of the wavefunction^29^ and the thermofield double purification of the density matrix. In both cases, we follow the dynamics using the time-dependent variational principle.

By bringing the study of many-body quantum chaos into contact with that of classical chaos, our approach opens up the full range of techniques available in the latter. For example, it allows the potential to examine how the classical KAM theorem for deformations from integrable behaviour and periodic orbits in classically chaotic systems may manifest in quantum systems^30–34^. It also suggests natural possibilities for efficient descriptions of late-time dynamics. This complementary perspective brings the study of quantum chaos full circle, recapitulating the characterisation of few particle quantum chaos through its projection to classically chaotic systems.

Our key results are, in the case of wavefunction MPS, we find a zero-parameter fit between the Lyapunov spectrum and the time-dependence of entanglement. In the thermofield MPS near the centre of the spectrum, we recover a semi-circular distribution of Lyapunov exponents for thermalising systems, as found previously in the case matrix models^35,36^, and a Gaussian distribution for integrable systems.

## Results

### Transverse-field Ising model in a longitudinal field

We apply the above reasoning to study the thermalisation of the Ising model with longitudinal and transverse fields:

1 $${\mathcal{H}} = \mathop {\sum}\limits_i {\left[ {J\sigma _i^{\mathrm{z}}\sigma _{i + 1}^{\mathrm{z}} + h^{\mathrm{z}}\sigma _i^{\mathrm{z}} + h^{\mathrm{z}}\sigma _i^{\mathrm{z}}} \right]} .$$

The properties of this model are well known; it is integrable when the longitudinal field *h*^z^ is zero and non-integrable otherwise. This allows us to investigate: (i) integrable systems (*J* = *O*(1), *h*^x^ = *O*(1) and *h*^z^ = 0), (ii) non-integrable/thermalising systems *J* = *O*(1), *h*^x^ = *O*(1) and *h*^z^ = *O*(1)) and (iii) nearly integrable systems *J* = *O*(1), *h*^x^ = *O*(1) and *h*^z^ ≪ *h*^x^). We apply the machinery of the time-dependent variational principle to determine trajectories, and the linearised time-dependent variational principle to determine Lyapunov spectra. Reflecting their different encodings of the relevant physics and different regimes of validity, we separate our discussions of the wavefunction MPS and thermofield MPS.

### Wavefunction MPS

**Fig. 1:**
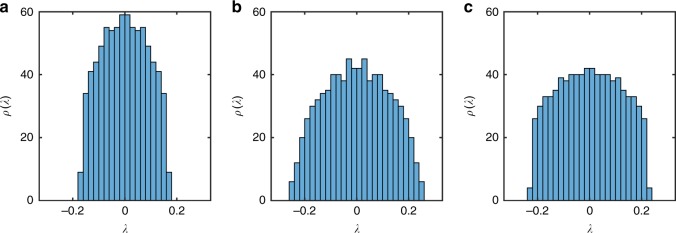
Lyapunov spectrum for a wavefunction MPS representation of Ising model dynamics. **a** Non-integrable case with *J* = 1, *h*^x^ = 0.5, *h*^x^ = 1. **b** Integrable case with *J* = 1, *h*^x^ = 0.5, *h*^x^ = 0. **c** Nearly Integrable case with *J* = 1, *h*^x^ = 0.5, *h*^x^ = 0.1. In all cases, the spectrum is obtained for an MPS representation of the wavefunction at bond order *D* = 20

We now consider the Lyapunov spectra evaluated from the wavefunction MPS starting from an initial product state |*ψ*(0)〉_*i*_ = (0.382 − 0.382*i*)|↑〉_*i*_ + (−0.595 + 0.595*i*)|↓〉_*i*_ near the bottom of the spectrum. The Lyapunov spectrum for the non-integrable, integrable and nearly integrable cases are shown in Fig. 1. All show a broad distribution of exponents, with no strong differences apparent between integrable and non-integrable cases. Although there is a difference apparent between the non-integrable and integrable or nearly integrable cases, this is insufficient to provide a diagnostic of integrability.

**Fig. 2:**
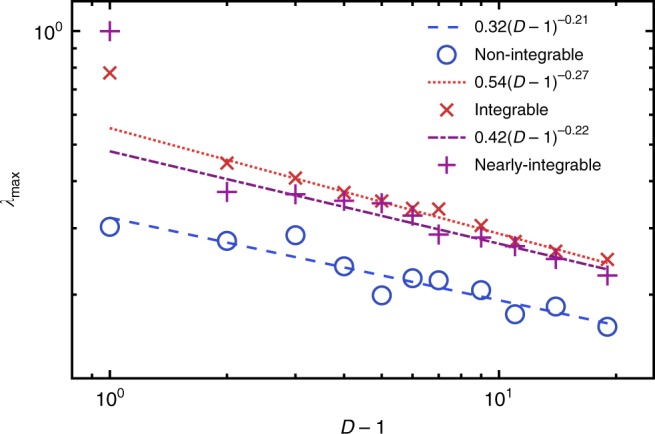
Maximum Lyapunov exponent versus bond order. The maximum Lyapunov exponent depends strongly upon the projection nonlinearities at different bond orders, tending to zero in the limit *D* → ∞. Here, we show the largest exponent varying with bond order for non-integrable (circles), integrable (crosses) and nearly integrable (pluses) systems. The largest exponent decreases like *λ*_max_(*D*) = 0.32(*D* − 1)^−0.21^ for non-integrable systems, *λ*_max_(*D*) = 0.54(*D* − 1)^−0.27^ for integrable systems and *λ*_max_(*D*) = 0.42(*D* − 1)^−0.22^ for nearly integrable systems

**Fig. 3:**
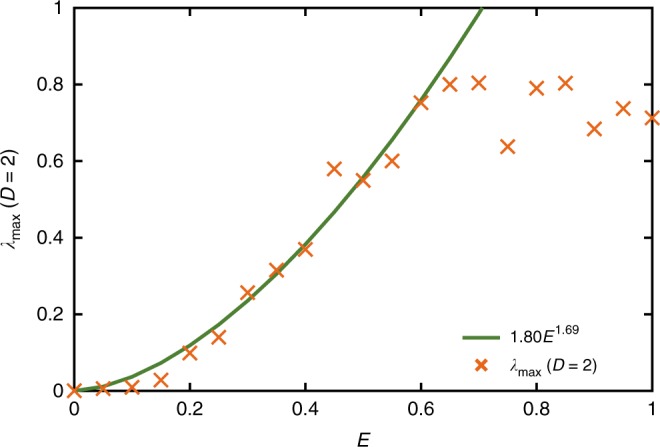
Maximum Lyapunov exponent versus energy density. It has previously been conjectured that *λ*_max_ ≤ 2*πk*_B_*T*/*ħ*, here, we observe that *λ*_max_ (*D* = 2) increases with energy density above the ground state, but appears to saturated at *E* ≈ 0.6. The initial growth of *λ*_max_ was fitted with a power law 1.80*E*^1.69^

A translationally invariant MPS is parametrised by a set of *D* × *D* matrices, *D* is often called the bond order. Since the nonlinearities and chaos of our dynamics arise from projection to the variational manifold, the Lyapunov spectrum varies with bond order. This situation is unlike the conventional use of matrix product methods, where increasing bond order gives increasingly accurate results. The dependence of the maximum Lyapunov exponent, *λ*_max_, upon *D* is shown in Fig. 2. This shows a decrease from *D* = 2 as *D* → ∞. The following discussion demonstrates the consistency of these results with physical observations. Note that in the translationally invariant case with spin 1/2, the projected dynamics is not chaotic at *D* = 1 by the Poincaré–Bendixson theorem, since the phase space is two-dimensional. The Lyapunov exponents are therefore zero in this case. Maldacena et al.^37^ have conjectured that the largest Lyapunov exponent of a quantum system has an upper bound related to its temperature *λ*_max_ ≤ 2*πk*_B_*T*/*ħ*;. The behaviour of *λ*_max_ for initial states of different energy can be seen in Fig. 3. At low energies, the exponent appears to increase as a power law before saturating at *E* ≈ 0.6.

**Fig. 4:**
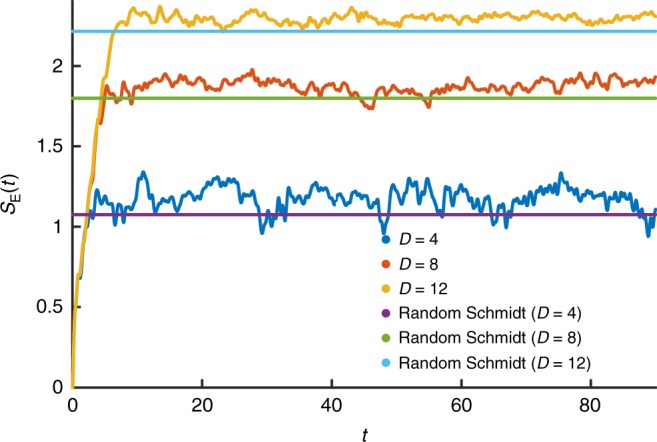
Entanglement entropy across a bond compared to randomly distributed Schmidt coefficients. At a given bond dimension, the entanglement entropy will saturate after a short time. The saturation value for the entanglement entropy is in strong agreement with a random uniform distribution of Schmidt coefficients

**Fig. 5:**
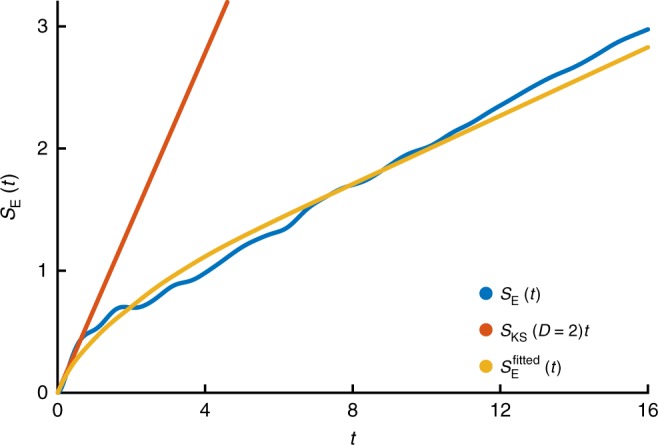
Entanglement entropy and Kolmogorov–Sinai entropy. The gradient of the entanglement entropy is determined by the Kolmogorov–Sinai entropy. The Kolmogorov–Sinai entropy at *D* = 2 accurately predicts the gradient of the entanglement entropy at *t* = 0 (orange). Substituting fitted forms for the Lyapunov spectrum and saturation entanglement into Eq. (4) gives a zero-parameter fit to the entanglement entropy (yellow). This fits may both be compared with the time evolution of *S*_E_(*t*) found using iTEBD at *D* = 100

The dependence of the entanglement entropy, *S*_E_ upon time is shown in Figs. 4 and 5. For a given bond order, *S*_E_ saturates. To a good approximation, this saturation value corresponds to drawing the Schmidt coefficients *s*_*n*_ from a distribution given by the modulus of the elements of a random *O*(*D*) vector. The mean Schmidt coefficients then correspond to $s_n = n\sqrt 6 /\sqrt {D(1 + D)(1 + 2D)}$, from which one may deduce a saturation entanglement at large bond order given by

2 $$S_{\mathrm{E}}^{{\mathrm{Sat}}}(D) = - \mathop {\sum}\limits_{n = 1}^D {s_n^2} \log s_n^2 \approx \log [0.65(D - 1) + 1].$$

With growing entanglement, the effective bond order of the quantum state (the bond order required for an accurate description) grows. We can use Eq. (2) to deduce this time-dependence; at the point where *S*_E_ crosses the bond order *D* saturation value, the bond order must be increased. A continuous approximation can be found by equating $S_{\mathrm{E}}^{{\mathrm{Sat}}}(D - 1) = S_{\mathrm{E}}(t)$, from which we obtain

3 $$D(t) = 1.54(e^{S_{\mathrm{E}}(t)} - 0.997) + 2.$$

As we discuss presently, this dependence of bond order upon time allow us to demonstrate the consistency of the Lyapunov spectrum and its variation with *D* with the physically relevant dependence of the entanglement entropy upon time.

The Kolmogorov–Sinai entropy *S*_KS_ is a measure of how quickly knowledge of a system’s initial state is lost in a chaotic system. It determines the growth rate of the volume of a region of phase space and, following Pesin’s theorem^38^, is given by the sum of the positive Lyapunov exponents.

Studies of single-particle quantum chaos have shown the relationship $\dot S_{\mathrm{E}}(t = 0) = S_{{\mathrm{KS}}}$, provided that starting wavefunction is as classical as possible^23,39,40^. Here, we find—as indicated in Fig. 5—that $\dot S_{\mathrm{E}}(t = 0) = S_{{\mathrm{KS}}}(D = 2)$. *D* = 2 corresponds to the most classical, non-trivial (recall that *D* = 1 has vanishing Lyapunov exponents) projected dynamics and is the many-body equivalent of the single-particle result. We speculate the following extension of this result:

4 $$\dot S_{\mathrm{E}}(t) = \frac{{S_{{\mathrm{KS}}}(D(t))}}{{(D(t) - 1)^2}}.$$

**Fig. 6:**
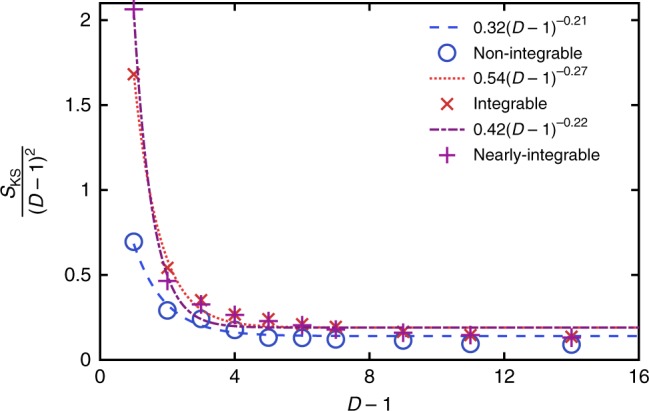
Kolmogorov–Sinai entropy versus bond order. The Kolmogorov–Sinai entropy scaled by (*D* − 1)^2^ is related to entanglement growth at short times. Here, we show the scaled KS entropy varying with bond order for non-integrable (circles), integrable (crosses) and nearly integrable (pluses) systems. The non-integrable KS entropy decreases like 0.14 + 1.6*e*^−1.08(*D*−1)^, the Integrable KS entropy decreases like 0.19 + 6.6*e*^−1.32(*D*−1)^ and the nearly integrable KS entropy decreases like 0.19 + 11.4*e*^−1.81(*D*−1)^

Our main justification is the very good, zero-parameter fit that it gives between our results for the entanglement and Lyapunov spectrum. A derivation may be possible from the entangled path integral^41^, where a similar result is obtained for the growth rate of bosonic fluctuations at a particular bond order. Figure 6 shows the Kolmogorov–Sinai entropy scaled by (*D* − 1)^2^ and its dependence upon bond order. At long times we expect *S*_E_(*t*) ~ *t* for thermalising systems, which using Eq. (4) suggests the fit

5 $$\frac{{S_{{\mathrm{KS}}}(D)}}{{(D - 1)^2}} = 0.14 + 1.6e^{ - 1.08(D - 1)}.$$

Combining Eqs. (3)–(5), the Lyapunov data imply a differential equation for *S*_E_(*t*) that we can integrate to find *S*_E_(*t*). Figure 5 shows the result plotted alongside entanglement obtained from a high bond-order iTEBD algorithm. The Lyapunov spectra underestimate the late-time linear growth rate of entanglement by about 15%. Note that Fig. 5 is plotted to times that extrapolate beyond times where our TDVP simulations are accurate.

It is apparent from these observations that the Lyapunov spectrum extracted from mapping the quantum dynamics of the wavefunction to classical Hamiltonian dynamics is not unique. There is no sense in which spectra collected in this way show numerical convergence, with increasing bond order. A moments reflection about the way in which the wavefunction MPS captures the physics of thermalisation shows why. At low bond order, the dynamics is very non-linear and thermalisation occurs via chaotic classical dynamics. Thermal averages are recovered in temporal averages of the simulated dynamics. As bond order increases, the MPS ansatz make better and better approximation to the underlying eigenstates and ultimately, thermalisation is captured in the same way as the conventional picture of eigenstate thermalisation. Thermal averages are obtained in instantaneous measurements after an initial period of dephasing reveals the intrinsic properties of the underlying eigenstates. However, the Lyapunov spectrum does have physical meaning. We have demonstrated how the physical quantity, *S*_E_(*t*), is related to the Lyapunov spectrum obtained on a sequence of variational manifolds.

### Thermofield MPS

The above analysis allows us to relate the chaos of projected quantum dynamics near to the edge of the spectrum to the process of thermalisation. We now apply our analysis of the Lyapunov spectrum to a matrix product-state representation of the thermofield double. The thermofield double represents the density matrix as a pure state in an enlarged Hilbert space [see the Methods section]. Expectations of operators are the same as calculated with the original density matrix. Techniques developed to study the dynamics of wavefunctions can then be adapted to effectively describe the dynamics of the density matrix. A particular benefit is that a matrix product-state approximation to the thermofield can efficiently describe both weakly entangled states and thermal states. It can therefore potentially be used both near the centre of the spectrum and at late times where the wavefunction MPS cannot. We consider an initial pure state near to the middle of spectrum, |*ψ*(0)〉_*i*_ = 0.448|↑〉_*i*_ + 0.873|↓〉_*i*_. The late-time dynamics of this are similar to the infinite-temperature state.

**Fig. 7:**
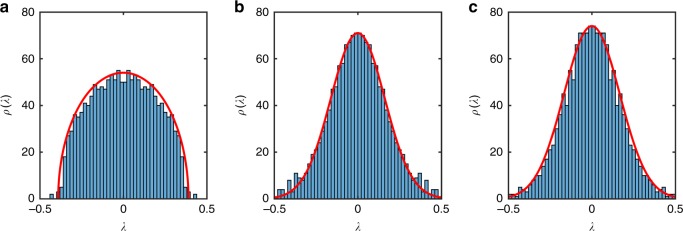
Lyapunov Spectrum for a thermofield MPS respresentation of Ising model dynamics. **a** Non-integrable case with *J* = 1, *h*^x^ = 0.5, *h*^z^ = 1. **b** Integrable case with *J* = 1, *h*^x^ = 0.5, *h*^z^ = 0. **c** Nearly integrable case with *J* = 1, *h*^x^ = 0.5, *h*^z^ = 0.1. In all cases, the spectrum is obtained for a wavefunction MPS at bond order ${\Bbb D} = 16$. The non-integrable case appears to fit a semi-circle distribution with radius *r* = 0.39, the integrable case appears to be Gaussian with standard deviation *σ* = 0.167 and the nearly integrable case appears to be Gaussian with standard deviation *σ* = 0.161

The Lyapunov spectra for the thermofield MPS dynamics are shown in Fig. 7. There is a clear distinction between the non-integrable, and integrable and nearly integrable cases. The former has a semi-circular distribution, whereas the latter are narrower and fit a Gaussian distribution (with long tails that have been cut-off in Fig. 7). For long-time averages, the Lyapunov spectrum for the nearly integrable case is expected to crossover from a pre-thermalisation Gaussian to a semi-circle distribution. There is a narrowing of the tails of our spectra at late times, but a clear demonstration of the emergence of a semi-circle is a subject for further study. The semi-circular distribution in the non-integrable case suggests a connection to random matrix theory. Such a connection has previously been explored in the context of matrix models^35,36^.

**Fig. 8:**
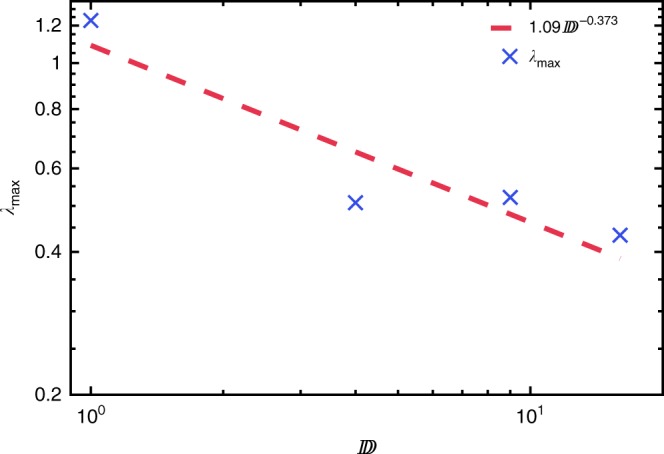
Maximum Lyapunov exponent vs. thermofield MPS bond order for the non-integrable system. The largest Lyapunov exponent for the Ising model with *J* = 1, *h*^x^ = 0.5, *h*^z^ = 1.0 obtained for an MPS representation of the thermofield double. The exponent appears to be approaching zero like $\lambda _{{\mathrm{max}}} = 1.09{\Bbb D}^{ - 0.373}$

Figure 8 shows the variation of the maximum Lyapunov exponent with bond order for the non-integrable case. Symmetry constraints that we impose upon the thermofield MPS tensor (See Supplementary Note 2C for details) restrict the bond order to ${\Bbb D} = 1,4,9,16$ etc., and together with the rapid growth of the number of Lyapunov exponents as $2(d^2 - 1){\Bbb D}^2$ this leads to rather few points in the figure. Note that since the dimension of the local Hilbert space is $d^2$, dynamical chaos occurs at ${\Bbb D} = 1$. Our numerics are fit by $1.09{\Bbb D}^{ - 0.373}$, or $1.17e^{ - 0.0173{\Bbb D}}$, but are also consistent with convergence $0.410 + 0.1740e^{ - 0.0116{\Bbb D}}$. The latter might be expected since the thermofield double (being a purification of the density matrix) encodes a limited set of observations corresponding roughly to a window of size $\frac{1}{2}{\mathrm{log}}_2{\Bbb D}$. When this window is larger than the correlation length, timescales of the dynamics are expected to converge to values characteristic of the observable thermalisation process.

**Fig. 9:**
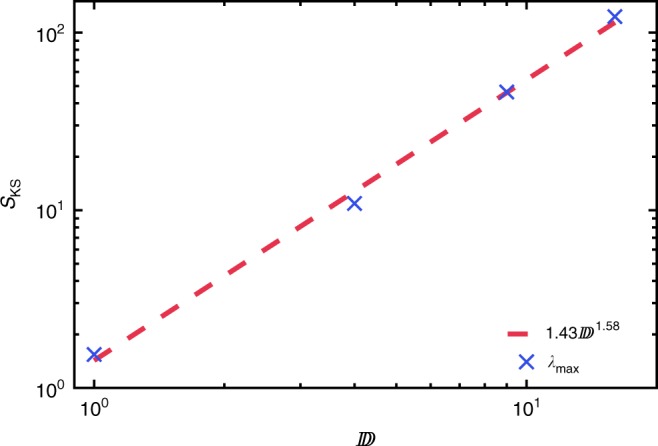
Kolmogorov–Sinai entropy vs. thermofield MPS bond order for non-integrable system. The Kolmogorov–Sinai entropy for the Ising model with *J* = 1, *h*^x^ = 0.5, *h*^z^ = 1.0 obtained for an MPS representation of the thermofield double. The KS entropy appears to be divering, growing like $S_{{\mathrm{KS}}} = 1.4270{\Bbb D}^{1.58}$

The Kolmogorov–Sinai entropy for the thermofield MPS is shown in Fig. 9. This is fit with $1.427{\Bbb D}^{1.58}$ to high accuracy. This scaling is less than ${\Bbb D}^2$ (the volume of phases space) of a typical classical dynamical system. This is consistent with unitary dynamics as ${\Bbb D}$ tends to infinity. Unlike wavefunction MPS, we have been unable to find a simple relationship between the Kolmogorov–Sinai entropy and thermofield entanglement.

## Discussion

The analysis presented above allows the thermalisation of local observables in a many-body quantum system to be recast as a set of chaotic classical Hamiltonian dynamics in two different ways: using the time-dependent variational principle to evolve MPS representations of the wavefunction and of the thermofield double. This picture is complementary to the dephasing of eigenstates in the conventional picture of eigenstate thermalisation and brings the study of quantum chaos full circle. Original studies of quantum chaos focussed upon single-particle quantum systems whose semiclassical limit is chaotic, or on many-body systems dominated by chaotic, single-particle dynamics^9–15^. The impact of this upon the level statistics provides a convenient way to discriminate between chaotic and non-chaotic behaviour that can be extended to many-body systems that do not have a clear semiclassical limit^17–20^. Our approach returns to a semiclassical analysis for many-body systems. Albeit, the semiclassical dynamics that we study describes entanglement structure whose origin is quantum mechanical. We have applied this to the Ising model with a longitudinal and transverse field using the time-dependent variational principle applied to matrix product states.

This analysis suggests a new relationship between the Komogorov–Sinai entropy (and its dependence upon bond order) and the entanglement, Eq. (4). This relationship holds not just for the initial entanglement growth but rather for the entire time-dependence of the entanglement. Using the thermofield MPS reveals a semi-circular distribution of Lyapunov exponents in the non-integrable case and Gaussian distribution in the integrable case. The former result has been anticipated in the context of gravitation^35,36^, where it was hypothesised that it may be universal. Calculating the Lyapunov spectra for finite systems is a natural extension of the present work and would enable comparison with calculations of the out-of-time ordered correlators^42–46^.

The exponential increase of data required to describe the dynamics of quantum systems presents an acute difficulty for efficient numerical simulation. The necessary data should decrease at late times for thermalising systems to allow for a simple description of the long-time dynamics. Whilst this decrease can be understood by dephasing, it is difficult to turn this insight into a practical scheme. Chaotic dynamics of classical projections give a possible route. Dynamical modes divide naturally into those that have revealed their chaotic nature on a given timescale and those that have not. The latter behave as quasi-regular modes and the former as a chaotic bath for them. This suggests a Langevin description, the crossover between early- and late-time dynamics being one between inertial and diffusive dynamics. An MPS Langevin equation can be constructed by adding noise and dissipation to the time-dependent variational principle and provides a suggestive link to random circuit analyses of thermalisation^44,47–54^. However, using the thermofield MPS may obviate the need for it. The thermofield MPS efficiently describes states at early and late times, and the time-dependent variational principle gives the appropriate dynamics. The remaining ingredient is to find a way of compressing the thermofield description at late times, and the multiple equivalent descriptions of an infinite-temperature state contains the seed of how this might be achieved (see Supplementary Note 2D).

Classical integrable systems show a remarkable robustness to perturbation. The KAM theorem shows that aspects of integrability remain through the presence of residual invariant tori (essentially periodic motions of action angle variables) when perturbations away from integrability are below some threshold. There has been speculation recently of whether such effects could be apparent in a quantum system^31^. It is inevitable that they are possible when quantum dynamics is projected to classical dynamics by observing on a finite window. This is a promising direction for future study, for example in the context of many-body localisation.

Matters of thermalisation and chaotic dynamics come to a head in quantum critical systems. These are the most rapidly dissipating and dephasing of quantum systems^55^, and it is no coincidence that recent years has seen their mapping to black holes—through the AdS/CFT correspondence—themselves the most rapidly scrambling (classically chaotic) of objects^56^. The semi-circle distribution of Lyapunov exponents that we have uncovered already makes links to works carried out in this context^35,36^. A direct application of MPS methods has limitations for the study of quantum criticality, however, because of diverging correlation lengths. It may be that other variational schemes such as MERA can do a better job, although in that case, dynamics are trickier. The view of quantum dynamics that we present should give an interesting complementary view of dynamical transitions observed after quenches and sweeps through quantum critical points.

To conclude, we have uncovered fundamental links between eigenstate thermalisation of many-body quantum systems and the chaos of related many-body classical systems, and demonstrated how techniques developed in the latter may be applied fruitfully to the study of fundamentally many-body quantum thermalisation.

## Methods

### Projecting quantum to classical dynamics

We have used two different methods to map quantum dynamics to classical Hamiltonian dynamics: by approximating both the wavefunction and the thermofield double purification of the density matrix by matrix product states and evolving them using the time-dependent variational principle. The numerical implementation of these two protocols is very similar—indeed, we use the same code (mutatis mutandis) for both cases—but both their regime of applicability and the manner in which they encode the physics is rather different.

A variational parametrisation of a system’s wavefunction picks out a sub-manifold of Hilbert space. The time-dependent variational principle projects quantum dynamics onto this manifold by mapping an updated quantum state—which in general lies outside of the manifold—onto the state on the manifold with which it has the highest fidelity. Remarkably this maps the Schrödinger equation to Hamiltons equations for a related classical system^24^. In particular, a quantity conserved by the exact dynamics will also be conserved by the projected dynamics, provided that the symmetry transformation generating it can be captured on the manifold. This permits sensible results to be obtained even at very long times^25^.

Consider a variational parametrization with a set of complex parameters {*X*_*i*_}. The time derivative of the wavefunction may be written $\partial _t|\psi \rangle \approx |\partial _{X_i}\psi \rangle \dot X_i$. It is tempting to substitute this into the Schrödinger equation, but the result is not correct since the action of the Hamiltonian on the state |*ψ*(**X**)〉 will generally take the state out of the variational manifold. Contracting with a tangent vector $\langle \partial _{\bar X_i}\psi |$ fixes this and permits us to write

6 $$\left\langle {\partial _{\bar X_i}\psi |\partial _{X_j}\psi } \right\rangle \dot X_j = i\left\langle {\partial _{\bar X_i}\psi |\hat{\mathcal{H}}|\psi } \right\rangle .$$

Using a particular basis for the tangent space, one may fix the Gramm matrix $\left\langle {\partial _{\bar X_i}\psi |\partial _{X_j}\psi } \right\rangle = \delta _{ij}$ after which identifying positions and momenta $q_i \equiv \sqrt 2 {\cal{I}}mX_i$ and $p_i \equiv \sqrt 2 {\cal{R}}eX_i$ reduces Eq. (6) to Hamilton’s equation for a classical system. Even though the parameters {*X*_*i*_} may quantify aspects of the entanglement structure of the wavefunction, they nevertheless provide a (semi-) classical description. This extends the notion of classical chaos considered in ref. ^57^ to semiclassical properties present even without the strict limit of $\hbar \to 0$. The technical details of applying this to matrix product states was developed in a seminal work of Jutho Haegeman et al.^24^. Application of the time-dependent variational principle to the wavefunction is standard. Details of our implementation are given in the Supplementary Note 2A and 2B.

The thermofield double^58^ is a purification of the density matrix. In the eigenbasis of the density matrix $\hat \rho = \mathop {\sum}\limits_\alpha {\gamma _\alpha } |\alpha \rangle \langle \alpha |$, it may be written as $|\psi{\hskip-4pt} \psi \rangle = \mathop {\sum}\limits_\alpha {\sqrt {\gamma _\alpha } } |\alpha \rangle \otimes |\alpha \rangle$, where $\gamma _\alpha$ are real positive weights that correspond to the Gibbs weights in thermal equilibrium, and $\alpha$ labels the eigenstates, $|\alpha \rangle$. Physical operators act on the first copy of the state only, so that expectations with the thermofield double are identical to those obtained from the density matrix: $\langle \psi{\hskip-4pt} \psi |\hat \theta |\psi {\hskip-4pt}\psi \rangle = Tr(\hat \rho \hat \theta )$. The time evolution of the thermofield double is determined by the Hamiltonian ${\cal{H}}{\hskip-6.8pt}{\cal{H}} = {\cal{H}} \otimes {\mathbf{1}} + {\mathbf{1}} \otimes {\cal{H}}$, which acts symmetrically on the doubled space. We also use a matrix product state parametrization of the thermofield double and evolve it using the time-dependent variational principle. The details of how to do this are given in the Supplementary Note 2C.

These two schemes for projecting quantum dynamics to classical Hamiltonian dynamics capture the physics in rather different ways and have different regimes of validity. The MPS approximation for a state is efficient near the top and the bottom of the spectrum. The bond order required to accurately describe a thermal state at temperature *T* scales as a double exponential^59^. The thermofield MPS is efficient both at the edges and near to the centre of the spectrum. These differences are also revealed in correlation lengths and the factorisation of averages such as $\langle \sigma _n^x\sigma _{n + N}^x\rangle$ for *N* greater than the thermal correlation length. The wavefunction MPS at low bond order captures such properties in explicit time averages. The instantaneous correlation length of the wavefunction MPS extracted from its transfer matrix^29^ can be longer than the thermal correlation length, reflecting the long-distance entanglement of its constituent eigenstates. The thermofield MPS captures the thermal correlation length in a rather different way. Since it is a purification of the density matrix, the thermofield MPS is directly related to observations and already includes the effects of dephasing.

### Lyapunov spectrum of projected dynamics

To extract the Lyapunov spectrum, we must characterise the divergence between nearby trajectories. Consider two trajectories both in the vicinity of a point on the MPS manifold with tensor $A_{ij}^\sigma$. Let these trajectories have parametrisations in terms of $X_{ij}^\sigma (t)$ and $X_{ij}^\sigma (t) + dX_{ij}^\sigma (t)$, respectively. Substituting each of these into the time-dependent variational principle Eq. (6) and subtracting, we obtain the following equation for the evolution of the difference between trajectories

7 $$\begin{array}{*{20}{l}} {d\dot X_{ij}^\sigma (t)} \hfill & = \hfill & {i\left\langle {\partial _{X_{ij}^\sigma }\partial _{X_{kl}^\gamma }\psi |\hat {\cal{H}}|\psi } \right\rangle dX_{kl}^\gamma (t)} \hfill \\ {} \hfill & {} \hfill & { + i\left\langle {\partial _{X_{ij}^\sigma }\psi |\hat {\cal{H}}|\partial _{X_{kl}^\gamma }\psi } \right\rangle d\bar X_{kl}^\gamma (t).} \hfill \end{array}$$

With the minor modification of allowing complex parameters, this equation is analogous to the linearised equations of motion used to extract the Lyapunov spectrum for classical trajectories. Similar structures have been used by Haegeman et al. in order to construct the excitation ansatz^60^, and form the zero-wavevector part of the kernel of a quadratic expansion of MPS path integral about its saddle point^41^. Extraction of the Lyapunov spectrum now proceeds as in the classical case, using Eq. (7) to find the instantaneous Lyapunov spectrum at each point along a trajectory given by Eq. (6) and averaging.

A final addition to this procedure—not usually used in calculating Lyapunov exponents for classical dynamical systems—is to parallel transport displacements between nearby trajectories along the variational manifold (see the Supplementary Note 3). This enables us to satisfy some constraints of projected quantum dynamics to numerical precision. The Lyapunov spectra of classical Hamiltonian systems are constrained by time-reversal invariance to have all of the exponents in positive/negative pairs with the same modulus. This property is inherited by the spectrum of projected quantum dynamics. An additional important property follows from using fidelity to determine the measure on the variational manifold. Fidelity is not changed by unitary time evolution. As a result, Lyapunov exponents calculated for unitary evolution must be identically zero. Evolution under a purely local Hamiltonian provides a useful test case, since it does not change the entanglement structure of a quantum state and the time-dependent variational principle Eq. (6) reproduces the full Schrödinger equation under projection onto any manifold. The Lyapunov exponents in this case must be identically zero.

## Supplementary information

Supplementary Information

## Data Availability

The datasets generated during and/or analysed during the current study are available from the corresponding author on reasonable request. The corresponding author can be contacted at andrew.hallam.10@ucl.ac.uk.

## Code availability

All code used to generate the Lyapunov spectra in this article is available at https://github.com/JGMorley/mps-miscellany.
