# Supplementary material for: The Lyapunov spectra of quantum thermalisation
Source: Nat Commun. 2019 Jun 20;10:2708. doi: 10.1038/s41467-019-10336-4 (PMC6586635; doi:10.1038/s41467-019-10336-4)
Supplement: Supplementary file 1 — Supplementary Information [file 41467_2019_10336_MOESM1_ESM.pdf]

The Lyapunov Spectrum of Quantum Thermalisation: Supplementary Information

A. Hallam *et al.*

SUPPLEMENTARY NOTE 1 - EXTRACTING THE CLASSICAL LYAPUNOV SPECTRUM

Here we provide some additional details of how to extract Lyapunov spectra from linearised equations of motion, describing the evolution of the displacement between neighbouring trajectories $\mathbf{X}(t)$ and $\mathbf{X}(t) + d\mathbf{X}(t)$. The asymptotic rate at which these two trajectories diverge (or converge) is characterized with a Lyapunov exponent. If the solution for this equation is $d\mathbf{X}(t) = Y(\mathbf{X}, t)d\mathbf{X}(0)$ then the Lyapunov exponent associated with these trajectories is

$$\lambda = \lim_{t\to\infty} \frac{1}{t} \log \frac{|d\mathbf{X}(t)|}{|d\mathbf{X}(0)|} = \lim_{t\to\infty} \frac{1}{t} \log (Y(\mathbf{X}, t)d\mathbf{X}(0)). \tag{1}$$

For almost all trajectories $\mathbf{X}(t)$ and almost all tangent vectors $d\mathbf{X}(t)$ the limit in Eq.(1) converges to the largest Lyapunov exponent of the system [11, 12].

Using a similar approach it is possible to calculate the entire Lyapunov spectrum. Instead of a single trajectory, consider a d-dimensional parallelepiped defined by d vectors tangent to the manifold at point $\mathbf{X}(t)$, $\mathbf{U}(t) = \{d\mathbf{X}^1(t), d\mathbf{X}^2(t), ..., d\mathbf{X}^d(t)\}$. The volume of the parallelepiped will evolve over time in a manner determined by the d Lyapunov exponents

$$\sum_{i=1}^{d} \lambda_i = \lim_{t\to\infty} \frac{1}{t} \log (\mathrm{Vol}^d(Y(\mathbf{X}, t)\mathbf{U}(0))). \tag{2}$$

Unfortunately, the Lyapunov spectrum cannot be easily extracted using this method. As $t \to \infty$ the different tangent vectors comprising the parallelepiped all begin to point in the direction of the largest Lyapunov exponent. Many methods have been used to circumvent this issue. We use an algorithm introduced by Bennetin et al. [1].

An orthonormal basis for the tangent space $\mathbf{V}(t) = \{d\hat{\mathbf{X}}^1(t), d\hat{\mathbf{X}}^2(t), ..., d\hat{\mathbf{X}}^d(t)\}$ is defined and then evolved for a short time:

$$\mathbf{U}(t + \delta t) = Y(\mathbf{X}, t)\mathbf{V}(t). \tag{3}$$

This evolution rotates and changes the length of each of the unit vectors in $\mathbf{V}(t)$. By performing a QR decomposition on $\mathbf{U}(t + \delta t)$ we can separate these two effects: $\mathbf{U}(t+\delta t) = \mathbf{Q}(t+\delta t)\mathbf{R}(t+\delta t)$. $\mathbf{V}(t+\delta t) \equiv \mathbf{Q}(t+\delta t)$ is a new orthonormal basis for the tangent space, obtained by rotating the basis vectors from the previous time step. Since $\det[U(t + \delta t)] = \prod_i R_{ii}$ the diagonal elements of $\mathbf{R}(t + \delta t)$ capture the extent to which the volume of the parallelepiped at the previous time step has changed.

This process is repeated iteratively to obtain a sequence of matrices $\mathbf{R}(t)$ from which me may extract the Lyapunov spectrum using

$$\lambda_i = \lim_{N\to\infty} \frac{1}{N\delta t} \sum_{n=1}^{N} \log |R_{ii}(n\delta t)|. \tag{4}$$

When studying finite systems it may be more convenient to calculate Lyapunov exponents using a time-series approach [3, 4, 8, 15]. This would involve extracting exponents from the evolution of observables, it is currently unclear if exponents can be accurately calculated in the quantum context using this approach.

SUPPLEMENTARY NOTE 2 - PROJECTING QUANTUM TO CLASSICAL DYNAMICS

Here we provide details of the time-dependent variational principle used to generate our numerical results. Our implementation closely follows that of Haegeman *et al*[5] and we refer to the original papers for further details. Here we give a brief summary noting in particular aspects that require modification for the thermofield MPS.

A. Matrix product state TDVP

A variational wavefunction $|\psi(A)\rangle$ defined by a matrix product state A^{σ}_{ij} evolves on the manifold of matrix product states according to Eq.(6) with the appropriate identification of varaibles and indices: $X \to A$, $i \to I \equiv \{i, j, \sigma\}$ giving

$$\langle \partial_{\bar{A}_I} \psi | \partial_{A_J} \psi \rangle \dot{A}_J = i \langle \partial_{\bar{A}_I} \psi | \hat{\mathcal{H}} | \psi \rangle. \tag{5}$$

Determining the time evolution of $|\psi(A)\rangle$ from equation Eq.(5) requires inversion of the Gram matrix $\langle \partial_{\bar{A}_I} \psi | \partial_{A_J} \psi \rangle$. In the case of matrix product states this is a $dD^2 \times dD^2$ matrix, however not all of the dD^2 tangent vectors are linearly independent so the Gram matrix cannot be inverted. As noted in Ref.[5], this can be resolved by imposing a gauge fixing condition on the states $|\partial_{A_J} \psi(A)\rangle$ parameterizing the tangent space. We follow Ref.[5] and use the *left tangent gauge fixing condition*, $\sum_{\sigma=1}^{d} A^{\sigma\dagger}_{ij} l_{jk} dA^{\sigma}_{kl} = 0$, where l is the left environment, *i.e.* the result of contracting the MPS state with its conjugate on every site to the left of a given site and dA is an update to the MPS tensor such that $A \to A + dA$. This gauge condition can be achieved by constructing $L_{i,(\sigma j)} = [A^{\sigma} l^{\frac{1}{2}}]_{ij}$ and calculating its null vectors, $[V_L])_{(i\sigma),j}$. If the null space is reshaped to $[V_L]^{\sigma}_{ij}$ then a dA that satisfies the tangent gauge condition can be written as

$$dA^{\sigma}(x) = l^{-\frac{1}{2}} V^{\sigma}_L X r^{-\frac{1}{2}}, \tag{6}$$

where r is the right environment.. Using this parameterizing the Gram matrix becomes diagonal and the time evolution of the state can determined by evaluating Eq.(5) to find the $(d^2 - 1)D \times D$ matrix X.

B. Inverse-free algorithm

While this algorithm is sufficient to determine the time evolution of a matrix product state at fixed bond dimen-

sion it has two flaws. Firstly, it necessarily involves inverting Schmidt coefficients and therefore encounters issues when a state has small Schmidt values. Secondly, there is no easy way to increase the bond dimension of the matrix product state as may be necessary if we start from say a product state initial state. Both of these issues can be solved by using an inverse-free version of the TDVP algorithm[6]. Here we provide minor modifications to this algorithm required for real-time evolution rather than imaginary-time evolution as studied in [6].

An inverse-free algorithm uses A in both left and right canonical forms, A_L and A_R respectively. For A_L the dominant left eigenvector of the transfer matrix is $l = \mathbb{I}$ and dominant right eigenvector is $r = cc^\dagger$. For A_R the dominant left eigenvector of the transfer matrix is $l = c^\dagger c$ and dominant right eigenvector is $r = \mathbb{I}$. The algorithm has three key steps:
i. A_R and c can be calculated from A_L in an inverse-free method by iterating

$$[c_{(i+1)}, A_{R(i+1)}] = RQ(A_L c_i) \tag{7}$$

until $c_{i+1} \approx c_i$ where $RQ(M)$ is an RQ decomposition.
ii. An inverse-free update of $A_L(t)$ is found by solving

$$min_{\tilde{A}_L} |\tilde{A}_L c(t+\delta t) - A_C(t+\delta t)| \tag{8}$$

where we have defined $A_C = A_L c = cA_R$, with
$A_C(t+\delta t) = A_L(t)c(t) + \delta t\ d(A_L c)/dt$
and $c(t+\delta t) = c(t) + \delta t\ dc/dt$. The time derivative of A_L is obtained from Eq.(5) and that of c from

$$(\mathbb{I} - \sum_{\sigma=1}^{d} A_L^\sigma \otimes \bar{A}_R^\sigma)\frac{dc}{dt} = \sum_{\sigma=1}^{d} \frac{A_L^\sigma}{dt} c A_R^{\sigma\dagger}. \tag{9}$$

Eq.(9) follows from writing $\frac{dc}{dt} = \frac{d}{dt}(\sum_{\sigma=1}^{d} A_L^\sigma c \bar{A}_R^\sigma)$ and using the right gauge fixing condition on A_R to impose $\sum_{\sigma=1}^{d} A_{ij}^\sigma c_{jk} d A_{R,kl}^{\sigma\dagger} = 0$. iv. Eq.(8) can be solved performing qr decompositions on $c(t+\delta t)$ and $A_C(t+\delta t)_{(\sigma i),j}$, $c(t+\delta t) = qr$ and $A_C(t+\delta t)_{(\sigma i),j} = QR$. We find $r = R$ so $A_L(t+\delta t)_{(\sigma i),j)} = Qq^\dagger$ and $A_L(t+\delta t)_{ij}^\sigma$ can be found by reshaping this matrix.

C. TDVP applied to the thermofield double

As we discuss presently, the MPS ansatz applied in the usual way efficiently describes states near to the top and bottom of the spectrum. States near to the centre of the spectrum we require an alternative variational parametrization. We use an MPS parametrization of the thermofield double. The thermofield double[13] is a purification of the density matrix. In the eigenbasis of the density matrix $\hat{\rho} = \sum_\alpha \gamma_\alpha |\alpha\rangle\langle\alpha|$, it may be written as $|\psi\!\!\!/\rangle = \sum_\alpha \sqrt{\gamma_\alpha}|\alpha\rangle \otimes |\alpha\rangle$, where γ_α are real positive weights that correspond to the Gibbs weights in thermal equilibrium, and α labels the eigenstates, $|\alpha\rangle$. Physical operators act on the first copy of the state only, so that expectations with the thermofield double are identical to those obtained from the density matrix: $\langle\psi\!\!\!/|\hat{\theta}|\psi\!\!\!/\rangle = Tr(\hat{\rho}\hat{\theta})$. The time-evolution of the thermofield double is determined by the Hamiltonian $\mathcal{H}\!\!\!\mathcal{H} = \mathcal{H} \otimes \mathbf{1} + \mathbf{1} \otimes \mathcal{H}$, which acts symmetrically on the doubled space.

Having identified the thermofield double and the appropriate Hamiltonian, we are free to construct an MPS ansatz for it and to evolve using the time-dependent variational principle. The time-dependent variational principle projects to the variational manifold by optimising the fidelity of the thermofield double. This amounts to optimising over a certain set of observations — specifically the trace-norm of the square root of the updated density matrix with the square root of its variational approximation. The square root guarantees that the fidelity is 1 for identical density matrices. The bond order of the MPS for the thermofield double does not have a direct interpretation in terms of the entanglement structure of individual states. Moreover, although evolution under $\mathcal{H}\!\!\!\mathcal{H}$ without approximation would preserve the purity of a sate, projection to the variational manifold takes pure states into mixed states. This is consistent with optimising over a certain set of observations, but quite different from the wavefunction MPS which remains pure. Although TDVP has been applied to the density matrix before [7], as far as we are aware, this is the first time that it has been used to follow real time evolution of a matrix product ansatz for it (though see [14] for a related work).

Several modifications are required to the apply MPS machinery to the thermofield double and its time evolution. We parametrize the thermofield double state $|\psi\!\!\!/(A\!\!\!A)\rangle$ by an expanded matrix product state $A\!\!\!A_{IJ}^{\sigma\delta}$ with a doubled physical index representing the two copies of the system. The thermofield double state is evolved using the expanded Hamiltonian, $\mathcal{H}\!\!\!\mathcal{H}$ The time-dependent variational principle Eq.(6) is modified accordingly with $A \to A\!\!\!A$, $\mathcal{H} \to \mathcal{H}\!\!\!\mathcal{H}$ and $\{i, j, \sigma\} \to \{I, J, \sigma, \delta\}$.

In order to obtain accurate results, we have made an important modification to the algorithm developed in [5] for MPS representations of the state. The thermofield double is evidently symmetrical between the two copies of the physical space; observations made on either copy will yield the same result. However, this is not necessarily reflected in an explicit symmetry of the tensor $A\!\!\!A_{I,J}^{\sigma\delta}$ and this can lead to the accumulation of numerical errors that break the symmetry. The MPS for the thermofield double can be written such that the symmetry between the two copies of the physical space is explicit. This is achieved for a bond order $D\!\!\!D = D^2$ thermofield MPS by imposing the symmetry $A\!\!\!A_{I,J}^{\sigma\delta} = A\!\!\!A_{\tilde{I},\tilde{J}}^{\delta\sigma}$ using an additional tangent space gauge fixing, where $I \equiv i \otimes i'$ and $\tilde{I} \equiv i' \otimes i$ with the indices $i, i', j, j' \in \{1, 2, ...D\}$. Note that a pure state with bond order D wavefunction MPS tensor A_{ij}^σ can be represented as a thermofield MPS of bond order $D = D\!\!\!D$ and tensor $A\!\!\!A_{ii',jj'}^{\delta\sigma} = A_{ij}^\sigma A_{i'j'}^\delta$.

In order to calculate our tangent state we find it more

convenient to work in the a slightly different gauge in which the symmetry condition is $\mathbb{A}^{\sigma\delta}_{I,J} = M_{IK}\mathbb{A}^{\delta\sigma}_{KL}M_{LJ}$, where

$$M = \begin{pmatrix} \mathbb{I}_{\frac{D}{2}(D+1)} & 0 \\ 0 & -\mathbb{I}_{\frac{D}{2}(D-1)}, \end{pmatrix} \tag{10}$$

The tangent gauge fixing is then achieved as follows: We first calculate $\mathbb{W}^{\sigma\delta}_{L,(IJ)}$ using the method described in Section A. Symmetric $(\frac{1}{2}W^{\sigma\delta}_{L,(I,J)} + M_{L,L'}\frac{1}{2}W^{\delta\sigma}_{L',(IJ)})$ and antisymmetric $(\frac{1}{2}\mathbb{W}^{\sigma\delta}_{L,(IJ)} - M_{LL'}\frac{1}{2}\mathbb{W}^{\delta\sigma}_{L',(I,J)})$ parts of W contribute separately to $d\mathbb{A}$ with corresponding symmetric and antisymmetric parts of the matrices X. The symmetrised and anti-symmetrised spaces are each smaller than the doubled space. A complete orthonormal basis for $\mathbb{W}$ is obtained by keeping the first $(d^2-1)D(D+1)/2 - D$ or $(d^2-1)D(D-1)/2 + D$ (where $\mathbb{D} = D^2$) columns of the Q from a QR decomposition of the symmetrised or anti-symmetrised $\mathbb{W}$ respectively. Full details of the implementation of this algorithm will be communicated elsewhere. This constraint also requires the modification of step iv. in the inverse-free algorithm. $\mathbb{A}_L(t+\delta t)$ is calculated using QR decompositions on $\mathbb{c}(t+\delta t)$ and $\mathbb{A}_C(t+\delta t)$ but the symmetry constraint requires Q to be modified. A new Q is obtained by performing a QR decomposition on the symmetrised $\frac{1}{2}Q_{((\sigma\delta)I),((\sigma\delta)'I')} + \frac{1}{2}MQ_{((\delta\sigma)\tilde{I}),((\delta\sigma)'\tilde{I}')}M^\dagger$ and keeping the first $\mathbb{D}$ columns.

The infinite temperature state takes a particularly simple and instructive form when represented in terms of a thermofield MPS. At $\mathbb{D} = 1$ it is given by $A^{\sigma\delta} = \delta^{\sigma\delta}/\sqrt{2}$. At $\mathbb{D} = D^2 > 1$ there are many ways to represent the state. A class of symmetrical thermofield MPS states can be constructed from a unitary matrix $U \in SU(dD)$ as

$$A^{\sigma\delta}_{IJ} = \frac{1}{\sqrt{d}}\sum_{\gamma=1}^{d} U_{(\sigma i),(\gamma j)}U_{(\delta i'),(\gamma j')}. \tag{11}$$

This follows from noting that i. the infinite temperature state is the same for any Hamiltonian and ii. that it is invariant under evolution with the Hamiltonian. Eq.(11) follows by representing an arbitrary time evolution of $A^{\sigma\delta} = \delta^{\sigma\delta}/\sqrt{2}$ with a bond operator representation of the time-evolution operator using the unitary U. This manifold of equivalent representations of the infinite temperature state resolves an apparent contradiction: on the one hand a state at the middle of the spectrum of a given Hamiltonian is expected to evolve towards the infinite temperature state, whilst on the other hand the projected dynamics is classically Hamiltonian and so cannot evolve to a single point in phase space. It also holds the seed of how to compress the thermofield MPS representation of a thermalising system at late times[9].

In supplementary figure 1 we see evidence of how this thermalization works in practise for thermofield double states. The *thermofield entanglement entropy* between sites of a state from the middle of the spectrum saturates at a value close to that obtained by averaging over the random unitary states defined in Eq.(11).

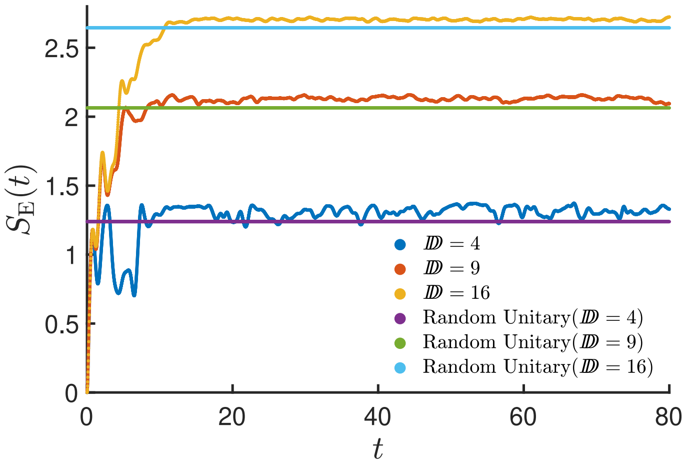

Supplementary Figure 1. *Entanglement of the midspectrum state:* The entanglement between sites of a Thermofield MPS state starting in the middle of the spectrum saturates at a value close to value obtained by averaging over random unitaries.

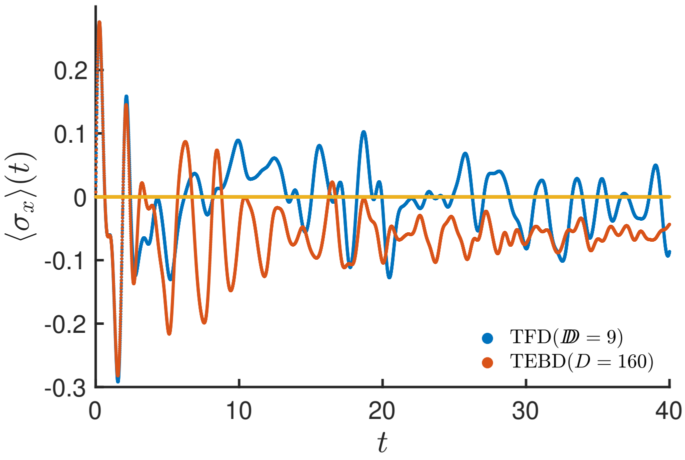

Supplementary Figure 2. *Thermal expectation values with the Thermofield MPS:* Starting from a product state in exactly the middle of the spectrum, the thermofield double ($\mathbb{D} = 9$) fluctuates around the correct thermal expectation value (yellow). It significantly outperforms a wavefunction MPS with much higher bond dimension ($D = 160$) evolved using time-evolving block decimation.

D. Comparing Classical Projections

These two schemes for projecting quantum dynamics to classical Hamiltonian dynamics capture the physics in rather different ways and have different regimes of validity. The MPS approximation for a state is efficient near the top and the bottom of the spectrum. The bond order required to accurately describe a thermal state at temperature T scales as a double exponential[2]. The thermofield MPS is efficient both at the edges and near to the centre of the spectrum. The latter is justified heuristically as follows: a thermofield MPS of bond order

$\mathbb{D}$ accurately describes observations up to a lengthscale $\sim \log_{d^2} \mathbb{D}$. If this is longer than the thermal correlation length in the final state, the description will accurately capture the dynamics. This occurs near the centre of the spectrum, where the effective temperature is high and the correlation length is short. In supplementary figure 2 we provide evidence that a thermofield MPS can outperform a wavefunction MPS of significantly higher bond dimension in the middle of the spectrum. The thermofield fluctuates around the correct thermal expectation value of a local observable while the wavefunction MPS does not.

These differences are also revealed in correlation lengths and the factorisation of averages such as $\langle \sigma^x_n \sigma^x_{n+N} \rangle$ for N greater than the thermal correlation length. The wavefunction MPS at low bond order captures such properties in explicit time-averages. The instantaneous correlation length of the wavefunction MPS extracted from its transfer matrix[10] can be longer than the thermal correlation length, reflecting the long-distance entanglement of its constituent eigenstates. The thermofield MPS captures the thermal correlation length in a rather different way. Since it is a purification of the density matrix, the thermofield MPS is directly related to observations and already includes the effects of dephasing. In this case, the instantaneous correlation length deduced from the transfer matrix is equal to the thermal correlation length and long distance correlators factorise in instantaneous observations.

SUPPLEMENTARY NOTE 3 - LYAPUNOV SPECTRUM OF PROJECTED DYNAMICS

In this section, we summarize how to extract Lyapunov spectra from projected quantum dynamics. The details of this are similar for our two projection schemes. For clarity, we will focus our discussion upon the wavefunction MPS, noting modifications necessary for the thermofield MPS as appropriate.

E. Distance on the Variational Manifold

As a first step to deducing the Lyapunov spectrum, we must assign a distance measure on the variational manifold. This is done using the fidelity between states with two different coordinates $\mathbf{X}$ and $\mathbf{X}+d\mathbf{X}$. As a simple example, consider a spin-1/2 coherent state given by

$$|\theta, \phi\rangle = e^{-i\phi/2} \cos \frac{\theta}{2} |\uparrow\rangle + e^{i\phi/2} \sin \frac{\theta}{2} |\downarrow\rangle.$$

The square of the distance between two such states $|\theta, \phi\rangle$ and $|\theta + d\theta, \phi + d\phi\rangle$ can be written, after expanding the fidelity between them to quadratic order, by

$$dS^2 = 1 - |\langle \theta, \phi | \theta + d\theta, \phi + d\phi \rangle|^2 = \frac{1}{4} \left(\sin^2 \theta d^2 + \phi d^2 \theta \right)$$

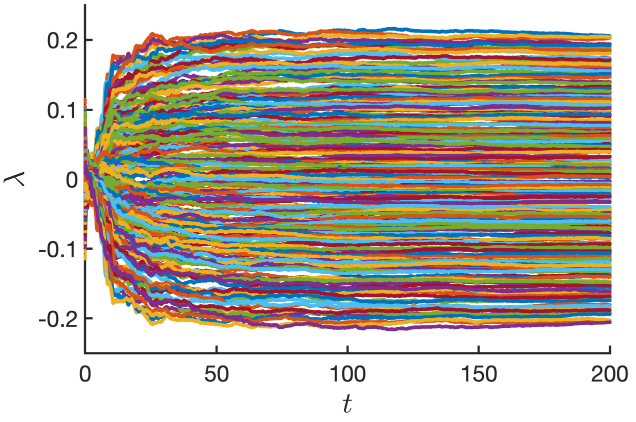

Supplementary Figure 3. *Convergence Plot for a Typical Thermalising System:* The time-averaged Lyapunov exponents *versus* time are shown for an MPS representation of the wavefunction of a typical thermalising system. We consider an Ising model with anti-ferromagnetic coupling $J = 1$, transverse field $h_x = 0.5$ and longitudinal field $h_z = 1$. The dynamics are obtained by integrating Eq.(5) and the spectrum from averaging instantaneous exponents obtained from Eq.(16) both using bond order $D = 10$.

corresponding to the usual distance measure on the Bloch sphere. In the case of translationally invariant states, we must use the fidelity density rather than fidelity, since the fidelity between translationally invariant states described by an MPS tensor A^σ_{ij} and one described by a tensor $A^\sigma_{ij} + dA^\sigma_{ij}$ scales as one over the total length of the system. As described in Sec. A, a small deviation from a translationally invariant MPS state described by a tensor A^σ_{ij} may be parametrised[5] in terms of freely-chosen complex tensor X^σ_{ij}: $A^\sigma_{ij} \to A^\sigma_{ij} + dA^\sigma_{ij}$. Surpressing auxiliary indicies for a moment for clarity, we can write $dA^\sigma = \sum_{\delta=1}^{d-1} l^{-1/2} V^{\sigma\delta} X^\delta r^{-1/2}$, where l and r are the left and right environments respectively, and $V^{\sigma\delta}_{ij} \equiv V^{\sigma\delta}_{ij}(A)$ is a tensor of null vectors to $A^{\sigma}_{ji}{}^*$ (reshaped into a matrix by pairing indices σ and j). This parametrisation was a crucial development of Haegeman *et al* in making the TDVP applied to MPS states tractable[5]. The distance measure takes a particularly simple form in terms of X: $dS^2 = \sum_{\sigma ij} X^{\sigma}_{ij}{}^* X^\sigma_{ji}$. This parametrisation is useful in determining the Lyapunov spectrum, the details of which we turn to next.

F. Linearised TDVP and the Lyapunov Spectrum

In Supplementary Note 1 we discussed how to calculate the Lyapunov spectrum of a trajectory in a dynamical system using vectors in its tangent space, in Supplementary Note 2 we explained how time evolution of a quantum state can be determined using the time dependent variational principle, we will now explain how to extract the Lyapunov spectrum of a quantum system using these

methods. We are interested in the evolution of the difference of two trajectories, *i.e.* the tangent vectors to the variational manifold. The equation of motion of these is given by linearizing Eq.(5) using the parametrization in terms of X given by Eq.(6);

$$d\dot{X}_a(t) = i\langle\partial_{\bar{X}_a}\partial_{\bar{X}_b}\psi|\hat{\mathcal{H}}|\psi\rangle dX_b(t) + i\langle\partial_{\bar{X}_a}\psi|\hat{\mathcal{H}}|\partial_{X_b}\psi\rangle d\bar{X}_b(t). \quad (12)$$

Our notation indicates a reshaping of the $(d^2-1)D \times D$ matrix X into a complex $(d^2-1)D^2$ vector. In the case of thermofield doubles, X is complex $(d(d+1)/2-1)D^2$-dimensional vector. The right hand side of Eq.(12) contains two parts: $F_1 = \langle\partial_{\bar{X}_a}\psi|\hat{\mathcal{H}}|\partial_{X_b}\psi\rangle$ is manifestly Hermitian and generates unitary rotations of the tangent vectors. $F_2 = \langle\partial_{\bar{X}_a}\partial_{\bar{X}_b}\psi|\hat{\mathcal{H}}|\psi\rangle$ is not Hermitian. Instead it is a symmetric matrix $F_2 = F_2^T$ and is responsible for the non-unitary evolution of tangent vectors.

The Lyapunov spectrum is calculated by measuring the extent to which a tangent vector $dX(t)$ has changed in magnitude between a time t and $t+\delta t$. Eq.(12) describes how the components dX_a transform but does not account for the transformation of the tangent space basis. This may be captured by allowing for parallel transport along the trajectory. Taking into account the parallel transport, Eq.(12) can now be written as

$$d\dot{X}(t) = \tilde{F}_1 dX(t) + \tilde{F}_2 d\bar{X}(t), \quad (13)$$

where F_1 and F_2 have been modified as follows:

$$F_1 \to \tilde{F}_1 = \langle\partial_{\bar{X}_b}\psi|\hat{\mathcal{H}}|\partial_{X_a}\psi\rangle - \Gamma^c_{ab}\dot{X}_c \quad (14)$$

$$F_2 \to \tilde{F}_2 = \langle\partial_{\bar{X}_a}\partial_{\bar{X}_b}\psi|\hat{\mathcal{H}}|\psi\rangle - \bar{\Gamma}^c_{ab}\dot{X}_c, \quad (15)$$

with $\bar{\Gamma}_{a,bc} = \langle\partial_{\bar{X}_b}\partial_{\bar{X}_c}\psi|\partial_{X_a}\psi\rangle$ and $\Gamma_{a,bc} = \langle\partial_{\bar{X}_b}\psi|\partial_{X_c}\partial_{X_a}\psi\rangle$ the Christoffel symbols for the variational manifold. With this modification we can calculate the Lyapunov spectrum. We separate the real and imaginary components of $dX = dX^R + idX^I$, $\tilde{F}_1 = \tilde{F}_1^R + i\tilde{F}_1^I$ and $\tilde{F}_2 = \tilde{F}_2^R + i\tilde{F}_2^I$. The real vector space is $2(d-1)D^2$ dimensional for matrix product states and $(d(d+1)/2-2)D^2$ for thermofield double states. Eq.(12) can be rewritten as:

$$\begin{pmatrix} d\dot{X}^R \\ d\dot{X}^I \end{pmatrix} = \begin{pmatrix} \tilde{F}_1^I + \tilde{F}_2^I & \tilde{F}_1^R - \tilde{F}_2^R \\ -\tilde{F}_1^R - \tilde{F}_2^R & F_1^I - \tilde{F}_2^I \end{pmatrix} \begin{pmatrix} dX^R \\ dX^I \end{pmatrix} \quad (16)$$

If $\tilde{F}_2 = 0$ the Hermitian property of $\tilde{F}_1$ would result in a totally antisymmetric matrix in Eq.(16), generating purely orthogonal rotations on the tangent vectors. $\tilde{F}_2$ is responsible for the changing magnitude of a tangent vector upon moving along a trajectory, and therefore for the generation of a non-zero Lyapunov spectrum. Local Hamiltonians $H = \sum_i h_i$ are important examples in which $\tilde{F}_2 = 0$. In this case, the parallel transport term cancels with F_2, guaranteeing that the Lyapunov spectrum is zero for all states. Having accounted for these details, the Lyapunov spectrum of the system can be calculated using Eq.(16) and the methods in Supplementary Note 1.

SUPPLEMENTARY REFERENCES

[1] G. Benettin, L. Galgani, A. Giorgilli, and J.-M. Strelcyn. Lyapunov characteristic exponents for smooth dynamical systems and for Hamiltonian systems - A method for computing all of them. I - Theory. II - Numerical application. *Meccanica*, 15:9–30, March 1980.

[2] Mario Berta, Fernando G. S. L. Brandão, Jutho Haegeman, Volkher B. Scholz, and Frank Verstraete. Thermal states as convex combinations of matrix product states. *Phys. Rev. B*, 98:235154, Dec 2018.

[3] R. Brown, P. Bryant, and H. D. I. Abarbanel. Computing the Lyapunov spectrum of a dynamical system from an observed time series. *Phys. Rev. A*, 43:2787–2806, March 1991.

[4] J. P. Eckmann, S. Oliffson Kamphorst, D. Ruelle, and S. Ciliberto. Liapunov exponents from time series. *Phys. Rev. A*, 34:4971–4979, Dec 1986.

[5] J. Haegeman, J. I. Cirac, T. J. Osborne, I. Pižorn, H. Verschelde, and F. Verstraete. Time-dependent variational principle for quantum lattices. *Phys. Rev. Lett.*, 107:070601, 2011.

[6] Jutho Haegeman, Christian Lubich, Ivan Oseledets, Bart Vandereycken, and Frank Verstraete. Unifying time evolution and optimization with matrix product states. *Phys. Rev. B*, 94(16):165116, 2016.

[7] Loïc Joubert-Doriol and Artur F Izmaylov. Problem-free time-dependent variational principle for open quantum systems. *The Journal of chemical physics*, 142(13):134107, 2015.

[8] H. Kantz. A robust method to estimate the maximal Lyapunov exponent of a time series. *Phys. Lett. A*, 185:77–87, January 1994.

[9] A. Hallam and A. G Green work in progress.

[10] Román Orús. A practical introduction to tensor networks: Matrix product states and projected entangled pair states. *Ann. Phys.*, 349:117–158, 2014.

[11] Valery Iustinovich Oseledec. A multiplicative ergodic theorem. liapunov characteristic number for dynamical systems. *Trans. Moscow Math. Soc.*, 19:197–231, 1968.

[12] David Ruelle. Ergodic theory of differentiable dynamical systems. *Publications Mathématiques de l'Institut des Hautes Études Scientifiques*, 50(1):27–58, 1979.

[13] Yasushi Takahasi and Hiroomi Umezawa. Thermo field dynamics. *Collect. Phenom.*, 2:55–80, 1974.

[14] Christopher David White, Michael Zaletel, Roger S. K. Mong, and Gil Refael. Quantum dynamics of thermalizing systems. *Phys. Rev. B*, 97:035127, Jan 2018.

[15] Alan Wolf, Jack B. Swift, Harry L. Swinney, and John A. Vastano. Determining lyapunov exponents from a time series. *Physica D*, 16(3):285 – 317, 1985.
